# MRI-based breast cancer radiogenomics using RNA profiling: association with subtypes in a single-center prospective study

**DOI:** 10.1186/s13058-023-01668-7

**Published:** 2023-06-30

**Authors:** Ah Young Park, Mi-Ryung Han, Bo Kyoung Seo, Hye-Yeon Ju, Gil Soo Son, Hye Yoon Lee, Young Woo Chang, Jungyoon Choi, Kyu Ran Cho, Sung Eun Song, Ok Hee Woo, Hyun Soo Park

**Affiliations:** 1 Department of Radiology, CHA Bundang Medical Center, CHA University, Seongnam, Republic of Korea; 2 Division of Life Sciences, College of Life Sciences and Bioengineering, Incheon National University, Incheon, Republic of Korea; 3 Department of Radiology, Korea University Ansan Hospital, Korea University College of Medicine, 123 Jeokgeum-ro, Danwon-gu, Ansan City, Gyeonggi-do 15355 Republic of Korea; 4 Division of Breast and Endocrine Surgery, Department of Surgery, Korea University Ansan Hospital, Korea University College of Medicine, Ansan City, Gyeonggi-do Republic of Korea; 5 Division of Hematology/Oncology, Department of Internal Medicine, Korea University Ansan Hospital, Korea University College of Medicine, Ansan City, Gyeonggi-do Republic of Korea; 6 Department of Radiology, Korea University Anam Hospital, Korea University College of Medicine, Seoul, Republic of Korea; 7 Department of Radiology, Korea University Guro Hospital, Korea University College of Medicine, Seoul, Republic of Korea

**Keywords:** Breast cancer, Radiogenomics, Magnetic resonance imaging, Molecular subtype, Texture analysis

## Abstract

**Background:**

There are few prospective studies on the correlations between MRI features and whole RNA-sequencing data in breast cancer according to molecular subtypes. The purpose of our study was to explore the association between genetic profiles and MRI phenotypes of breast cancer and to identify imaging markers that influences the prognosis and treatment according to subtypes.

**Methods:**

From June 2017 to August 2018, MRIs of 95 women with invasive breast cancer were prospectively analyzed, using the breast imaging-reporting and data system and texture analysis. Whole RNA obtained from surgical specimens was analyzed using next-generation sequencing. The association between MRI features and gene expression profiles was analyzed in the entire tumor and subtypes. Gene networks, enriched functions, and canonical pathways were analyzed using Ingenuity Pathway Analysis. The *P* value for differential expression was obtained using a parametric *F* test comparing nested linear models and adjusted for multiple testing by reporting *Q* value.

**Results:**

In 95 participants (mean age, 53 years ± 11 [standard deviation]), mass lesion type was associated with upregulation of *CCL3L1* (sevenfold) and irregular mass shape was associated with downregulation of MIR421 (sixfold). In estrogen receptor-positive cancer with mass lesion type, *CCL3L1* (21-fold), SNHG12 (11-fold), and MIR206 (sevenfold) were upregulated, and MIR597 (265-fold), MIR126 (12-fold), and *SOX17* (fivefold) were downregulated. In triple-negative breast cancer with increased standard deviation of texture analysis on precontrast T1-weighted imaging, *CLEC3A* (23-fold), *SRGN* (13-fold), *HSPG2* (sevenfold), *KMT2D* (fivefold), and *VMP1* (fivefold) were upregulated, and *IGLC2* (73-fold) and *PRDX4* (sevenfold) were downregulated (all, *P* < 0.05 and *Q* < 0.1). Gene network and functional analysis showed that mass type estrogen receptor-positive cancers were associated with cell growth, anti-estrogen resistance, and poor survival.

**Conclusion:**

MRI characteristics are associated with the different expressions of genes related to metastasis, anti-drug resistance, and prognosis, depending on the molecular subtypes of breast cancer.

**Supplementary Information:**

The online version contains supplementary material available at 10.1186/s13058-023-01668-7.

## Background

Breast cancer is a complex disease that consists of heterogeneous molecular subtypes [1]. Clinically, the subtypes are divided into luminal, HER2-enriched, and triple-negative breast cancer (TNBC) based on immunohistochemical staining of the estrogen receptor (ER), progesterone receptor, and human epidermal growth factor receptor 2 (HER2) status [2–4]. In recent decades, the management strategies of breast cancer based on molecular subtypes have resulted in better treatment outcomes than anatomical staging [1]. However, treatment effectiveness and prognosis still vary from patient to patient and are unpredictable. Furthermore, drug resistance occurs frequently: anti-endocrine resistance is reported in more than 30% of patients with luminal type cancer, and chemoresistance is reported in about 50% of patients with TNBC [1, 5]. More recently, high-throughput gene sequencing techniques have been rapidly evolving and it was shown that even the same breast cancer subtype can display various genetic alterations and lead to different therapeutic effects and prognosis.

Radiogenomic investigation of breast cancer can help us better understand tumor characteristics at the gene level and provide imaging markers to help select optimal treatment and predict prognosis more precisely. It aims to correlate quantitative and qualitative imaging phenotypes with gene mutation or expression [6]. There have been several retrospective radiogenomic analyses using MRI in breast cancer since 2012. They revealed that tumor size, lesion type, shape, or heterogeneous enhancement at contrast-enhanced T1-weighted imaging correlated with genetic changes related to cell cycle, recurrence, or tumor microenvironment [7–9]. However, few prospective studies have correlated clinically accessible MRI features with whole RNA-sequencing data. In addition, few studies have focused on the potential imaging markers for treatment decision and prognosis prediction specific to molecular subtypes. We hypothesized that MRI characteristics of breast cancer might reflect genetic alternations associated with tumor prognosis and treatment response according to histological subtypes.

Therefore, the purpose of the present study was to correlate the qualitative and quantitative MRI phenotypes of breast cancer (regarding tumor morphology and heterogeneity) and the whole RNA-sequencing data, and thereby to identify imaging surrogates that could be useful to predict clinical outcomes and determine management strategies based on the ER and HER2 status of cancer. We assessed tumor morphology using the breast imaging-reporting and data system (BI-RADS) lexicon and tumor heterogeneity using texture analysis [10].

## Methods

### Study participants

**Fig. 1:**
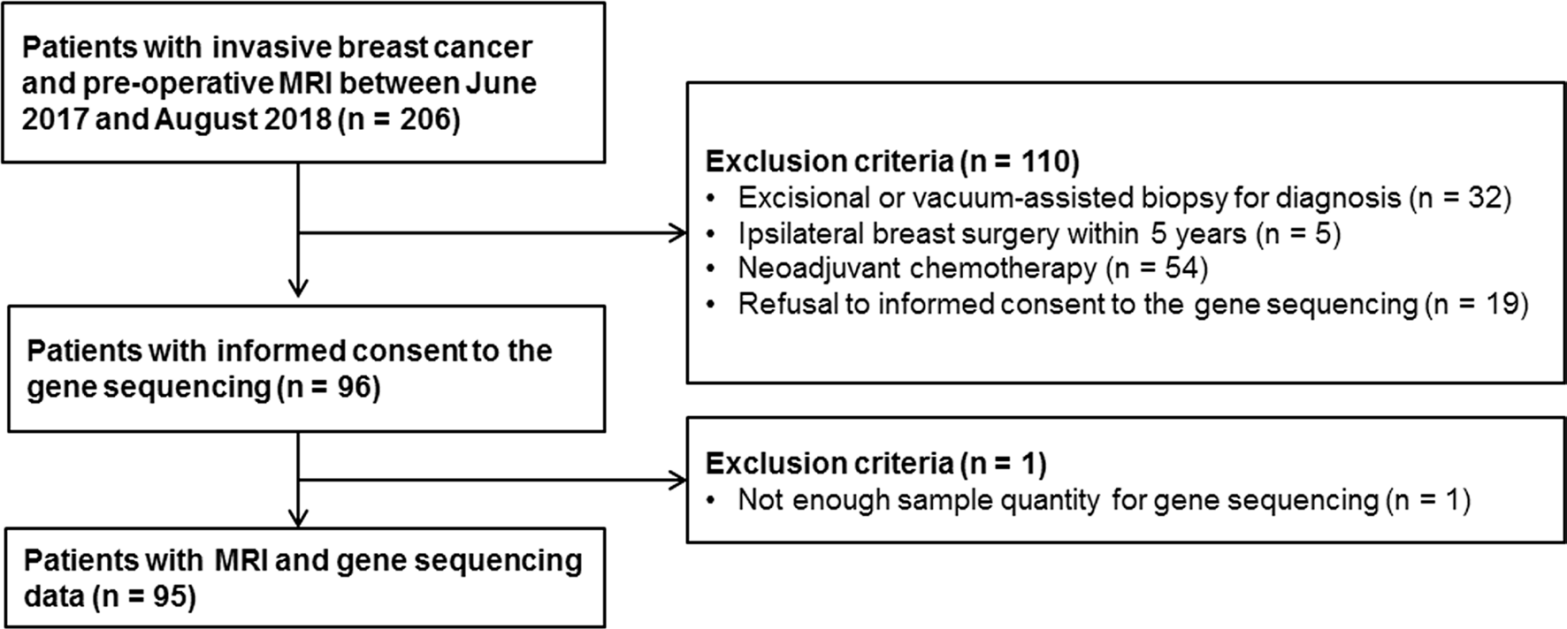
Flowchart of study participants

From October 2017 to August 2018, 206 consecutive participants who had pathologically proven invasive breast cancer underwent breast MRI before treatment at Korea University Ansan Hospital. Of the 206 participants, 111 were excluded for at least one of the following reasons: (a) excisional or vacuum-assisted biopsy for diagnosis (*n* = 32); (b) ipsilateral breast surgery within 5 years (*n* = 5); (c) neoadjuvant chemotherapy (*n* = 54); (d) refusal of informed consent to the gene sequencing (*n* = 19); and (e) insufficient sample quantity for RNA testing (*n* = 1). Ultimately, 95 participants with a total of 95 invasive breast cancers were included in our study (Fig. 1). Next-generation sequencing was performed using the whole genomic RNA obtained from surgical specimens. This prospective study was approved by the institutional review board of Korea University Ansan Hospital (approval no. 2017AS17145 and 2021AS0318) and written informed consent was obtained from all participants. This work was supported by Basic Science Research Program through the National Research Foundation of Korea (NRF) funded by the Korea government (Ministry of Science, ICT and Future Planning [No. NRF-2021R1A2C1010565, No. NRF-2020R1C1C1012288, and No. NRF-2020R1G1A1102372]).

### MRI acquisition

We used a 3 T MRI system (MAGNETOM Skyra; Siemens Healthineers, Erlangen, Germany) with a dedicated 4-channel breast coil. Bilateral transverse fat-suppressed T2-weighted two-dimensional turbo spin-echo imaging (repetition time msec/echo time msec, 4050/56; matrix, 307 × 384; field of view, 340 × 340 mm; flip angle 120°; reconstruction voxel size, 0.44 × 0.44 × 3 mm; slice thickness, 3 mm) and transverse fat-suppressed T1-weighted three-dimensional volumetric interpolated breath-hold examination (3.44/1.36; matrix, 320 × 320; field of view, 320 × 320 mm; reconstruction voxel size, 1 × 1 × 1 mm; slice thickness, 1 mm) were performed. Precontrast T1 mapping was generated using two different flip angles (2°, 9°) in the transverse plane encompassing the entire tumor volume before dynamic imaging was performed. Postcontrast images were acquired after gadoterate meglumine (Uniray; Dongkook Life Science Co., Ltd, Seoul, Korea) was injected intravenously at a dose of 0.2 mL/kg of body weight, followed by a 30-mL saline flush. Five postcontrast series were obtained, at 93, 180, 268, 356, and 443 s after the start of contrast agent injection.

### MRI analysis

**Fig. 2:**
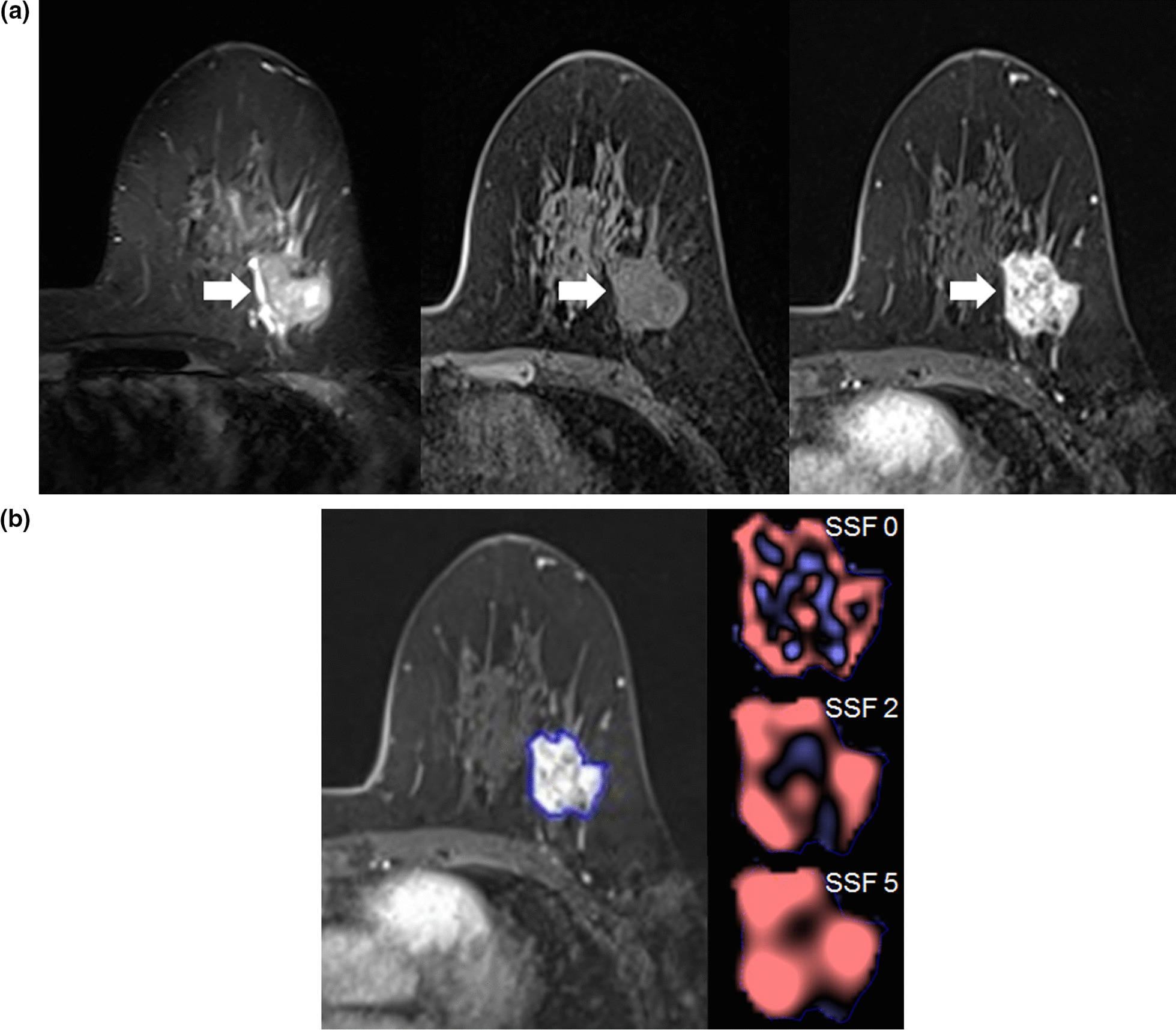
Invasive ductal carcinoma in a 61-year-old-woman. **A** Tumor morphology assessments were performed on T2-weighted MRI, and pre-and postcontrast T1-weighted MRI using the BI-RADS lexicon. An irregularly shaped, marginated, and heterogeneous enhancing mass (arrows) is seen. **B** Texture analysis was performed within a region of interest using SSFs of 0 (unfiltered texture), 2 (fine-filtered texture), and 5 (coarse-filtered texture)

Image evaluation was performed by two radiologists (B.K.S. and A.Y.P., with 20 and 7 years of experience in breast MRI, respectively) who achieved consensus. They were blind to histological finding. Tumor morphology was evaluated according to the BI-RADS lexicon [10]. Tumor heterogeneity was evaluated with commercially available software after manual segmentation of a whole tumor and assessed using a filtration histogram technique, a first-order statistical-based texture analysis (TexRAD; Feedback Medical Ltd., Cambridge, UK) (Fig. 2). A total of 62 qualitative and quantitative MRI phenotypes were extracted.

In morphology, the lesion type was divided into mass or non-mass enhancement [10]. In masses, shape, margin, and internal enhancement characteristics were evaluated and in non-mass enhancement, distribution, and internal enhancement patterns were evaluated according to the BI-RADS [10]. For texture analysis, a region of interest along the entire enhancing tumor margin was drawn on the postcontrast T1-weighted images obtained at the first phase of contrast injection. The same was applied to the precontrast T1-weighted and T2-weighted images. After tumor segmentation, texture features were extracted according to various spatial scale filters (SSFs). Each SSF corresponded to the same number of millimeters of pixel scales: unfiltered texture (SSF 0), fine texture (SSF 2, 2 mm), and coarse texture (SSF 5, 5 mm). The following six texture parameters were extracted based on the gray-level intensity histogram at SSF 0, 2, and 5 on each sequencing: mean pixel intensity, standard deviation (SD), mean of positive pixels (average gray-level intensity above zero threshold), entropy (irregularity or complexity of gray-level distribution), kurtosis (pointiness of the histogram), and skewness (asymmetry of the histogram) [11]. Thus, a total of 54 quantified texture parameters were obtained and dichotomized by the mean value for the 95 cancers according to the previous studies [12–15].

### Histologic analysis

Histologic analysis of breast cancer was performed according to the World Health Organization’s classification [16]. The immunohistochemical staining results for ER and HER2 were evaluated. ER was considered positive when the Allred score was three or higher. HER2 expression was considered positive when the score was 3+ in immunohistochemistry, or 2+ in immunohistochemistry and HER2 gene amplification in silver in situ hybridization.

### RNA sequencing and analysis

For RNA sequencing, mRNA-seq libraries were prepared using a paired-end sequencing sample preparation kit (TruSeq RNA Access library kit [Illumina, Inc., San Diego, CA, USA]). Indexed libraries were then submitted to an Illumina NovaSeq6000, and the paired-end (2 × 100 base pair) sequencing was performed.

Trimming of low-quality and adapter sequences from paired-end reads was conducted using Trim Galore software (version 0.6.5; https://www.bioinformatics.babraham.ac.uk/projects/trim_galore/) with Cutadapt (version 1.15) [17]. In short, the Spliced Transcripts Alignment to a Reference 2-pass method was used to align trimmed reads to the human reference genome (hg19) [18]. Then, Spliced Transcripts Alignment files produced in the above step were processed using Picard tools to add read group information, sort, mark duplicates, and index.

Identified networks were ranked by the score calculated for each network according to the fit of the network to the set of focus genes on the Ingenuity Pathway Analysis. We conducted two different methods to measure the significance of the association between differentially expressed genes with a *Q* value < 0.1 and the canonical pathway (generalized pathways that represent common properties of a particular signaling module or pathway): (1) the ratio of the number of molecules from the selected genes that map to the pathway to the total number of molecules that map to the canonical pathway; and (2) the *P* value calculated using Fisher’s exact test to determine the probability that the association between the selected genes and the canonical pathway can be explained by chance alone.

### Statistical analysis

Differential expression of individual genes between the two groups of each MRI phenotype was analyzed using Tablemaker (version 2.1.1) and Ballgown R package (version 2.22.0) [19]. Fragments per kilobase of transcript per million mapped reads were used to estimate the gene expression level. The *P* value for differential expression was extracted using a parametric *F* test comparing nested linear models. The Ballgown stattest function was used to calculate the log twofold change (log2FC) of the gene expression between two groups of each MRI phenotype of all tumors. The same analysis was performed for the subgroups according to ER and HER2 status. Functional enrichment and canonical pathway analysis were performed using the Ingenuity Pathway Analysis software (Ingenuity Systems, Redwood City, CA, USA). The *P* value was adjusted for multiple testing by reporting *Q* value, a statistical method for estimating false discovery rate, and a *Q* value < 0.1 was used to select differentially expressed genes. Finally, differential gene expression results were visualized using a volcano plot and heat map in R. The Plot function in R and R package “calibrate (version 1.7.7)” was used to generate a volcano plot. The heat map was visualized using R packages “ggplot2 (version 3.3.3)” and “made4 (1.64.0)”.

## Results

### Study participant characteristics

We included 95 patients (all women; mean age, 53 years ± 11 SD) with 95 breast cancers (mean size, 24.9 mm ± 13.0). Lesion characteristics are summarized in Table 1, and the MRI phenotypes are shown in Additional file 1: Table S1.

**Table 1:** Study participants characteristics

Characteristics	Values
Age (years)	53 ± 10 (25–81)
Lesion size (mm)	24.9 ± 13.1 (6–72)
Histologic type	
Invasive ductal carcinoma	81
Mucinous carcinoma	4
Invasive lobular carcinoma	3
Invasive micropapillary carcinoma	3
Tubular carcinoma	2
Medullary carcinoma	1
Metaplastic carcinoma	1
Molecular subtype	
Luminal	67
HER2-enriched	13
Triple-negative	15

Values mean number of cancers or mean data ± standard deviation (range)

### Differentially expressed genes according to MRI phenotypes in all cancers

In 95 tumors, 18 genes were expressed differentially according to the three MRI phenotypes with the standard of *Q* < 0.1, *P* < 0.05 and log2FC > 2.0 or < − 2.0: three genes were upregulated and 15 were downregulated. Among 18 differentially expressed genes, three were protein-coding genes, five were noncoding genes, and ten were pseudogenes or unidentified genes. Table 2 summarizes eight differentially expressed protein-coding and noncoding genes according to MRI phenotypes.

**Table 2:** Differentially expressed genes associated with breast cancer according to MRI phenotypes in all tumors

MRI phenotype	Genes	*Q* value	Log2FC	*P* value
Lesion type	*CCL3L1*	0.063	2.81	0.001
	SNORA31	0.053	2.77	< 0.001
	SNORA45	0.047	2.81	< 0.001
Mass shape	LINC01124	0.001	− 2.09	< 0.001
	Y-RNA	0.005	− 2.13	< 0.001
	MIR421	0.005	− 2.57	< 0.001
	*DEGS1*	0.003	− 2.66	< 0.001
	*VIMP*	0.096	− 2.76	0.001

There were 8 genes that were significantly upregulated (log2FC > 2.0) or downregulated (log2FC < − 2.0) with of *P* < 0.05 and *Q* < 0.1 according to the MRI phenotypes in our study. Pseudogenes or unidentified genes were not included

Breast cancer with mass lesion type showed the upregulation of *CCL3L1* (log2FC = 2.81; *P* = 0.001), compared with non-mass enhancement type. In mass lesions, irregular shape showed the downregulation of MIR421 (log2FC = − 2.57; *P* < 0.001).

### Differentially expressed genes according to MRI phenotypes in cancer subtypes

Table 3 and Additional file 2: Table S2 summarizes differentially expressed genes according to MRI phenotypes based on the ER and HER2 status (*Q* < 0.1, *P* < 0.05 and log2FC > 2.0 or < − 2.0).

**Table 3:** Summary of differentially expressed genes associated with breast cancer according to MRI phenotypes in histologic subgroups

Histologic subgroup	MRI phenotype	Genes	*Q* value	Log2FC	*P* value
ER-positive cancer	Lesion type	*CCL3L1*	0.047	4.40	0.002
		SNHG12	0.066	3.43	0.002
		MIR206	0.053	2.86	0.002
		*SLC39A7*	0*.*048	2.65	0.002
		*CD9*	0*.*080	2.04	0.003
		*SOX17*	0.003	− 2.28	< 0.001
		MIR126	< 0.001	− 3.63	< 0.001
		MIR597	0.001	− 8.05	< 0.001
HER2-positive cancer	Mean of positive pixels on PostcontrastT1 (SSF 2)	*MLKL*	0*.*064	2.20	< 0.001
	Mean of positive pixels on T2 (SSF 5)	*CXCL10*			
	*0.*080	− 3.27	< 0.001		
Triple-negative cancer	Standard deviation on PrecontrastT1 (SSF 5)	*CLEC3A*	*0.*036	4.50	0.001
		*SRGN*	0*.*062	3.72	0.001
		*HSPG2*	0*.*084	2.85	0.002
		*ABCC5*	0.007	2.36	< 0.001
		*KMT2D*	0.035	2.35	< 0.001
		*FBP1*	0.035	2.29	< 0.001
		*VMP1*	0.037	2.26	0.001
		*FZD2*	0.085	2.06	0.002
		*PRDX4*	0.094	− 2.80	0.002
		*IGLC2*	0.016	− 6.18	< 0.001

There were 143 genes that were significantly upregulated (log2FC > 2.0) or downregulated (log2FC < − 2.0) with of *P* < 0.05 and *Q* < 0.1 according to the MRI phenotypes in subgroup analysis (Additional file 2, Table S2). Pseudogenes or unidentified genes were not included. Among them, the 20 genes in this Table 3 were reported to be relevant to breast cancer in previous literatures [20–27, 33–43, 45, 46]

**Fig. 3:**
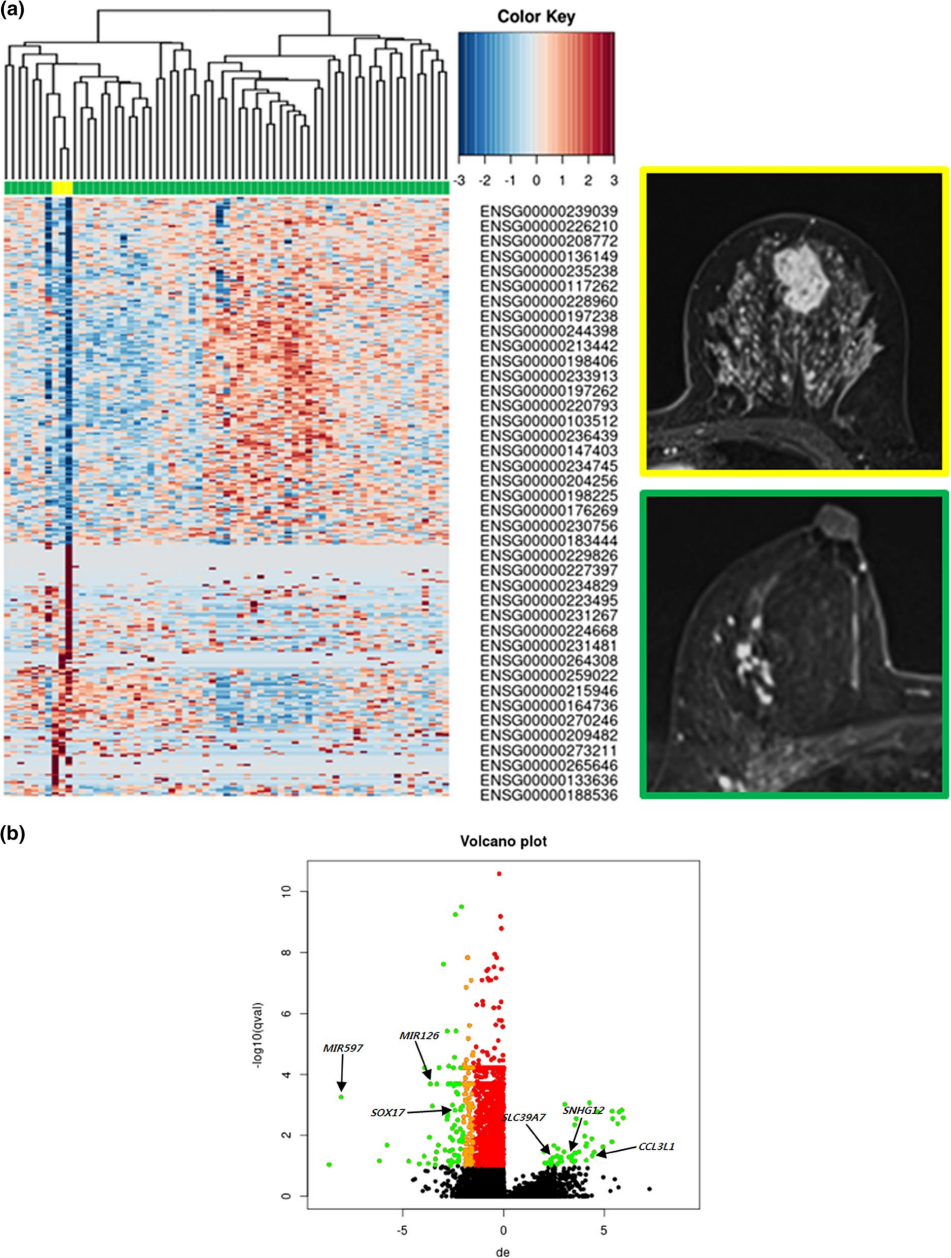
Radiogenomic correlations according to the lesion type in 65 participants with ER-positive breast cancer. **A** A heat map image demonstrates 320 differentially expressed genes according to the lesion type (*P* < 0.05 and log2FC > 2.0 or < − 2.0). Columns represent 320 individual differentially expressed genes (Ensemble Gene ID). The color key indicates the degree of differential gene expression in either direction to upregulation (red) or downregulation (blue). A MRI image framed in yellow shows breast cancer with mass type in a 36-year-old woman. The mass shows irregular shape, irregular margin, and heterogeneous enhancement. A MRI image framed in green shows breast cancer with non-mass enhancement type in a 52-year-old woman. This lesion shows focal homogenous non-mass enhancement. **B** A volcano plot demonstrates the differentially expressed genes in breast cancers with mass type compared with those with non-mass enhancement (*Q* < 0.1). The x-axis represents the degree of differential gene expression (log2FC) of individual genes, and the y-axis represents the negative logarithm of their *Q* value to base 10. Positive log2FC values represent upregulation in cancers with mass type compared with those with non-mass enhancement, and negative values represent downregulation. Green circles represent differentially expressed genes between cancers with lesion type mass and cancers with non-mass enhancement with *Q* < 0.1 and log2FC > 2.0 or < − 2.0

In 65 ER-positive breast cancers, mass type showed the upregulation of 31 genes and the downregulation of 22 genes, compared with non-mass enhancement type (Additional file 2: Table S2): *CCL3L1* (log2FC = 4.4; *P* = 0.001), *SNHG12* (log2FC = 3.43; *P* = 0.002), MIR206 (log2FC = 2.86; *P* = 0.002), *SLC39A7* (log2FC = 2.65; *P* = 0.002), and *CD9* (log2FC = 2.04; *P* = 0.003) were upregulated. MIR126 (log2FC = − 3.63; *P* = 0.001), MIR597 (log2FC = − 8.05; *P* < 0.001), and *SOX17* (log2FC = − 2.28; *P* < 0.001) were downregulated. Figure 3 shows gene expression data using a heat map and volcano plots according to lesion type.

**Fig. 4:**
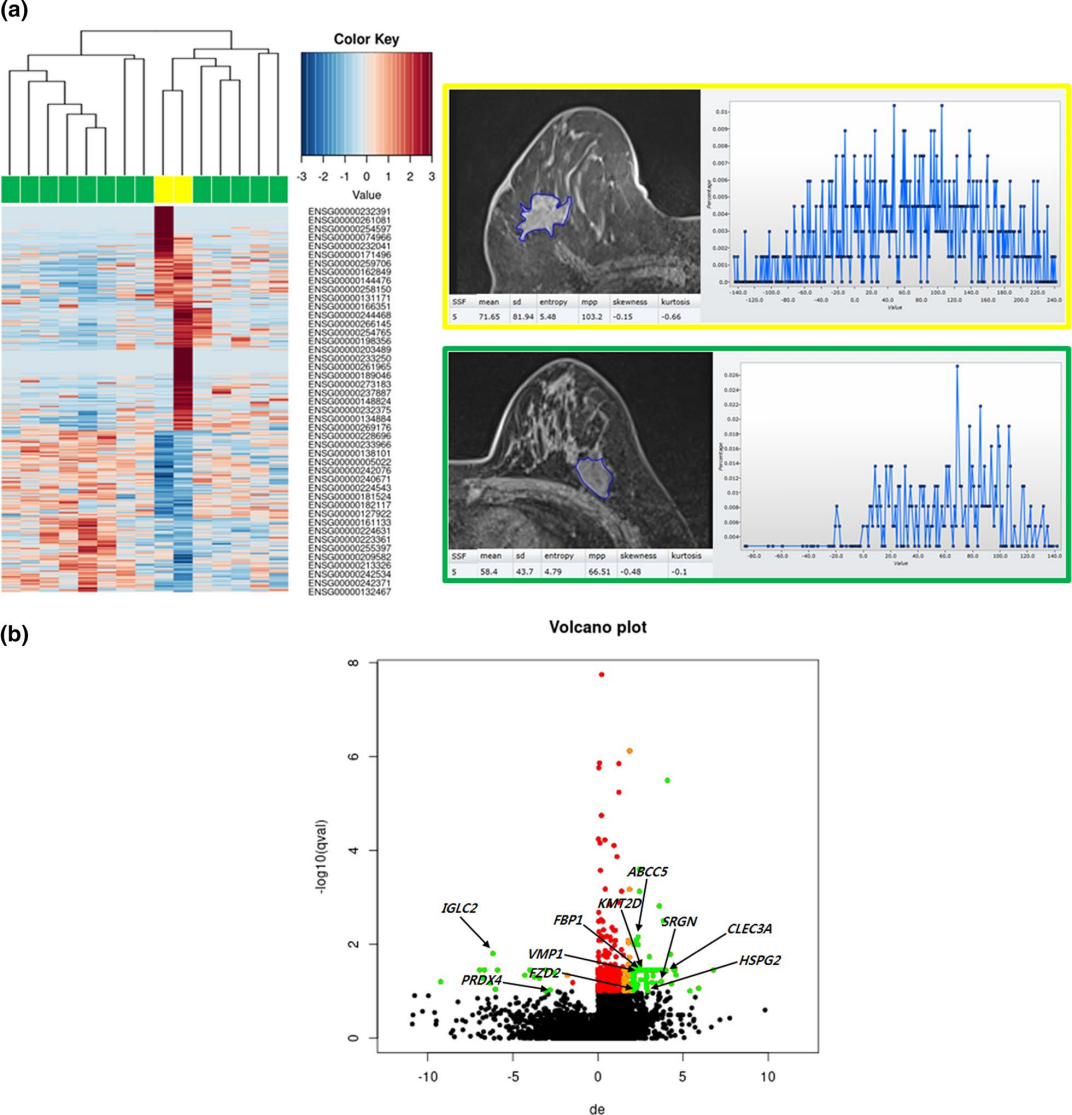
Radiogenomic correlations according to SD on PrecontrastT1 at SSF5 in 15 participants with TNBC. **A** A heat map image demonstrates 536 differentially expressed genes according to the lesion type (*P* < 0.05 and log2FC > 2.0 or < − 2.0). Columns represent 536 individual differentially expressed genes (Ensemble Gene ID). The color key indicates the degree of differential gene expression in either direction to upregulation (red) or downregulation (blue). The MRI image and histogram framed in yellow shows breast cancer with increased SD on PrecontrastT1 at SSF5 (81.9, > mean value 63.9) in a 48-year-old woman. The MRI image and histogram framed in green shows breast cancer with decreased SD on PrecontrastT1 at SSF5 (43.7, ≤ mean value 63.9) in a 50-year-old woman. **B** A volcano plot demonstrates the differentially expressed genes in breast cancers with increased SD on PrecontrastT1 at SSF5 compared with those with decreased SD on PrecontrastT1 at SSF5 (*Q* < 0.1). The x-axis represents the degree of differential gene expression (log2FC) of individual genes, and the y-axis represents the negative logarithm of their *Q* value to base 10. Positive log2FC values represent upregulation in cancers with mass type compared with those with non-mass enhancement, and negative values represent downregulation. Green circles represent differentially expressed genes between cancers with increased SD on PrecontrastT1 at SSF5 and cancers with decreased SD on PrecontrastT1 at SSF5 with *Q* < 0.1 and log2FC > 2.0 or < − 2.0

In 15 TNBC, tumors with increased SD on PrecontrastT1 at SSF5 showed upregulation of 29 genes and downregulation of 14 genes, compared with those with decreased SD (Additional file 2: Table S2): *CLEC3A* (log2FC = 4.5; *P* < 0.001), *HSPG2* (log2FC = 2.85; *P* = 0.002), *KMT2D* (log2FC = 2.35; *P* = 0.001), *VMP1* (log2FC = 2.26; *P* < 0.001), *SRGN* (log2FC = 3.72; *P* < 0.001), *ABCC5* (log2FC = 2.36; *P* < 0.001), *FBP1* (log2FC = 2.29; *P* < 0.001) and *FZD2* (log2FC = 2.06; *P* = 0.002) were upregulated. Genes for *IGLC2* (log2FC = − 6.18; *P* < 0.001) and *PRDX4* (log2FC = − 2.8; *P* = 0.002) were downregulated. Figure 4 shows gene expression data using a heat map and volcano plots according to SD on PrecontrastT1 at SSF5.

In 16 HER2-positive cancers, tumors with increased mean of positive pixels on PostcontrastT1 at SSF 2 showed the upregulation of *MLKL* (log2FC = 2.2; *P* < 0.001), compared with those with decreased mean of positive pixels (Table 3). HER2-positive tumors with decreased mean of positive pixels on T2 at SSF 5 showed upregulation of *CXCL10* (log2FC = 3.27; *P* < 0.001), compared with those with increased mean of positive pixels on T2 at SSF 5.

### Gene network, enriched functions, and canonical pathway

**Fig. 5:**
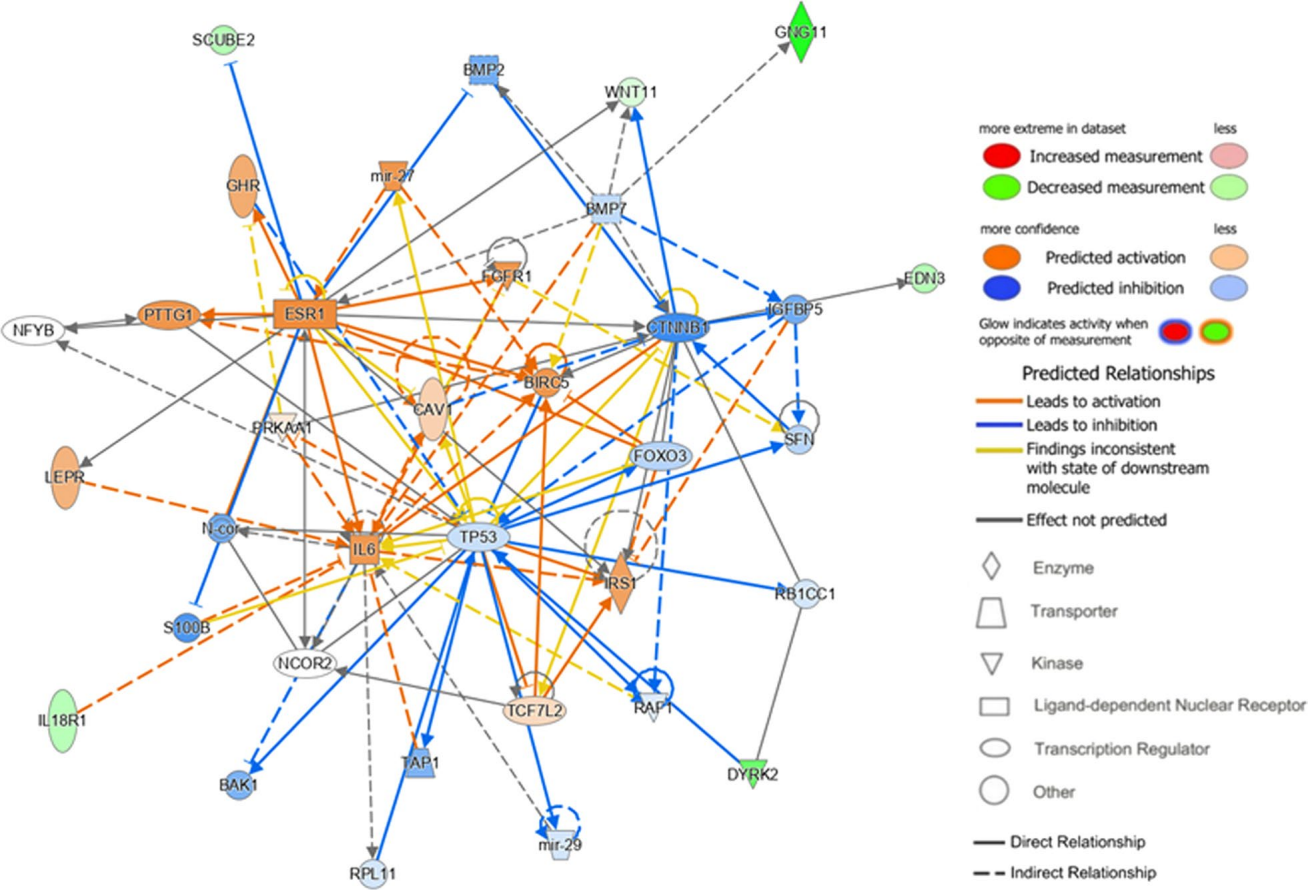
The top network by Ingenuity Pathway Analysis using differentially expressed genes (*Q* < 0.1) according to the lesion type in ER-positive cancer. *ESR1*, *BIRC5*, *CAV1*, *FGFR1*, *IL6*, *MIR27*, and *PTTG1* were upregulated with direct or indirect interactions between them. The top functions of this network included cell cycle, cellular growth and proliferation with a score of 11. The network is presented graphically by nodes (gene–gene products) and edges (biological interactions between nodes). The shape of the nodes indicates the functional class of the gene product, and the node color intensity indicates the degree of up- (red or orange) and down- (green or blue) regulation. ER = estrogen receptor, *ESR* = Estrogen Receptor 1, *BIRC5* = Baculoviral IAP Repeat Containing 5, *CAV1* = Caveolin 1, *FGFR1* = Fibroblast Growth Factor Receptor 1, *IL6* = Interleukin 6, *MIR27* = MicroRNA 27a, *PTTG1* = PTTG1 Regulator of Sister Chromatid Separation, Securin

Gene network analysis was performed using the differentially expressed genes of each MRI phenotype (*Q* < 0.1). In one of the top networks for the lesion type in ER-positive cancers, *ESR1*, *BIRC5*, *CAV1*, *FGFR1*, *IL6*, MIR27, and *PTTG1* were upregulated (Fig. 5). The top functions of this network included cell cycle, cellular growth, and proliferation, with a score of 11.

The functions associated with breast cancer were annotated primarily concerning the lesion type in ER-positive cancers (Table 4). Mass-type ER-positive cancers showed enriched functions of cell division, migration, transition, anoikis, and chemotaxis of breast cancer cell lines. In the canonical pathway, mass-type ER-positive cancers showed the activation of the STAT3 pathway.

**Table 4:** Enriched functional annotation of the differentially expressed genes according to lesion type in ER-positive Cancer

Functional annotation	*P* value	Genes
Cell division of breast cancer cell lines	0.018	*IGF1*
Migration of breast cancer cell lines	0.022	*DPP4,IGF1,SLC16A4,WNT11*
Transition of breast cancer cell lines	0.023	*IGF1*
Arrest in G0/G1 phase transition of breast cancer cell lines	0.032	*IGF1*
Breast or gastric cancer	0.035	*ACACB,ADRA1D,CRHBP,DPP4,DYRK2,FER1L5,HMX1,IFNA10,IGF1,IL1RL1,LARP7,MAP6,SCN3A,SCN7A,SCUBE2,SLC16A4,WNT11*
Breast or gynecological cancer	0.037	*ACACB,ADRA1D,CRHBP,DPP4,DYRK2,FER1L5,IGF1,IL18R1,IL1RL1,LARP7,MAP6,RCVRN,SCN3A,SCN7A,SCUBE2,SLC16A4,WNT11,ZNF136*
Breast or ovarian carcinoma	0.037	*ACACB,ADRA1D,CRHBP,DPP4,FER1L5,IGF1,IL1RL1,MAP6,SCN3A,SCN7A,SCUBE2,WNT11*
Anoikis of breast cell lines	0.038	*IGF1*
Mitogenesis of breast cancer cell lines	0.041	*IGF1*
Entry into S phase of breast cancer cell lines	0.046	*IGF1*
Chemotaxis of breast cancer cell lines	0.047	*IGF1*

Enriched functional annotations were obtained from Ingenuity Pathway Analysis (Ingenuity Systems, Redwood City, Calif) with an input of differentially expressed genes according to MRI phenotype (*Q* < 0.1) and the functions associated with breast cancer or general cancer were annotated

## Discussion

Several MRI-based radiogenomic breast cancer studies have shown associations between imaging features and genetic variation, but few prospective studies on molecular subtypes have been conducted. In a single-center prospective cohort, we evaluated associations between the MRI morphological and texture characteristics and whole RNA-sequencing data from 95 breast cancers based on ER and HER2 status. We found that lesion type and mass shape were associated with eight differentially expressed genes. In subgroup analysis, lesion type in ER-positive cancers and various texture features, including SD on precontrast T1-weighted images in TNBCs, mean on early postcontrast T1-weighted images, and mean of positive pixels on T2-weighted images in HER2-positive cancers, were associated with differential gene expressions related to metastasis, anti-drug resistance, and survival.

In all tumors, morphologic features, such as lesion type and mass shape, showed associations with differential gene expression. In mass-type cancers, *CCL3L1* was upregulated sevenfold compared with non-mass enhancement cancers*.* CCL3L1 is a chemokine expressed in lymphocytes and interacts with CCR5 in breast cancer cells, which promotes the migration and invasion of breast cancer cells [20]. In mass–type cancer, irregular shape cancer was associated with a sixfold downregulation of MIR421, compared with oval- or round-shape cancer. MIR421 is associated with metastasis and recurrence [21]. We are considering all mass types of breast cancer, particularly those with oval or round masses, which may be more aggressive than cancers with non-mass enhancement. A qualitative radiogenomic study by Woodard et al. [9] showed that the non-mass enhancement revealed by MRI was associated with lower recurrence scores, which is in line with our observation.

In ER-positive cancers, *CCL3L1*, *SNHG12*, and MIR206 were upregulated 21-fold, 11-fold, and sevenfold, respectively, in mass–type cancers, compared to non-mass enhancement–type cancers. These genes promote breast cancer cell proliferation, migration, and invasion [20, 22, 23]. CD9 was also upregulated 4.1-fold in mass-type cancer, which is associated with the chemoresistance to doxorubicin and 5-fluorouracil [24]. In contrast, MIR597, MIR126, and *SOX17* were downregulated 265-fold, 12-fold, and fivefold, respectively. These genes act as tumor suppressors in breast cancer, and their low expression was associated with a high TNM stage and shorter overall survival [25–27]. Therefore, the lesion type can be an important imaging surrogate in ER-positive cancers, and mass lesion type may indicate a poor prognosis. In addition, gene network analysis regarding to mass-type ER-positive cancers showed that *ESR1*, *BIRC5*, *CAV1*, *FGFR1*, *IL6*, MIR27, and *PTTG1* were upregulated. *ESR1* mutation and *FGFR1* overexpression cause anti–hormone resistance in ER-positive cancers [28, 29]. Overexpression of *BIRC5*, CAV1, and MIR27 is associated with nodal metastasis and shorter survival [30, 31]. Especially in mass-type ER-positive cancers, the STAT3 pathway was activated in the canonical pathway analysis, which suggests the increased potential of metastasis in ER-positive cancers [32]. Therefore, our results suggest that ER-positive cancers presented as masses may require active monitoring and treatment.

In TNBC, increased SD on PrecontrastT1 at SSF 5 was a significant MRI feature associated with genes for chemoresistance, metastasis, and shorter survival. SD on PrecontrastT1 means a variation of the signal intensity for the tumor before contrast injection, which indicates the degree of the inherent heterogeneity of the tumor. TNBC with increased SD on PrecontrastT1 showed a 23-fold, 13-fold, sevenfold, fivefold, and fivefold upregulation of *CLEC3A*, *SRGN*, *HSPG2*, *KMT2D*, and *VMP1* respectively, and these genes are associated with poor survival in breast cancer [33–37]. Recent studies elucidated that *HSPG2* is a promising drug target for metastatic TNBC, and *SRGN* regulates metastasis and promotes chemoresistance to 5-Fluorouracil in TNBC [34, 35]. In addition, TNBC with increased SD on Precontrast T1 at SSF 5 showed a 5–onefold, 4.9-fold, and 4.2-fold upregulation of *ABCC5*, *FBP1*, and *FZD2*. These genes are associated with metastasis, increased chemoresistance or decreased chemosensitivity [38–41]. Meanwhile, *IGLC2* and *PRDX4* were downregulated 73-fold and sevenfold, respectively, and their high expression is associated with better disease-specific or relapse-free survival in breast cancer [42, 43]. According to a recent study by Lee et al. [44], SD of histogram-based texture analysis on PrecontrastT1 was one of the important MRI parameters predicting prognostic markers in breast cancer, and its importance in the prediction was higher with coarse filtration (SSF3–5) than with no or fine filtration (SSF 0–2). Thus, this was consistent with our results. The evaluation of SD on PrecontrastT1 using MRI texture analysis may play an important role in predicting disease prognosis and selecting candidates for the potential targeted therapy for the genes above.

In HER2-positive cancers, increased mean on PostcontrastT1 at SSF 2 was associated with a fivefold upregulation of *MLKL*. This implies that a high degree of early enhancement representing tumor angiogenesis could be associated with the increased proliferative activity and large size of HER2-positive cancer [45]. Meanwhile, the decreased mean of positive pixels on T2 at SSF 5 was associated with a tenfold upregulation of *CXCL10*. This suggests that a low T2 signal intensity representing high tumor cellularity of tumor is associated with cell proliferation, invasion, and immune cell infiltration in HER2-positive cancer [46].

Our study suggests several key advances in the radiogenomics of breast cancer. First, we performed a prospective study using the BI-RADS lexicon and histogram-based texture analysis. This study suggests that easily accessible MRI features can reflect the altered expressions of individual genes or cancer-related pathways. Second, we found associations between MRI phenotypes and gene expression differences in subgroups based on ER and HER2 status. This suggests that certain MRI features in each subgroup of breast cancer may imply specific genetic alterations that may serve as potential targets for treatment. From this perspective, our results could be used to build radiogenomic models of breast cancer for precision medicine.

There are some limitations to this study. First, it was conducted at a single institution in a single nation. Second, we excluded invasive breast cancers that received neoadjuvant chemotherapy; thus, we could not show the radiogenomic features of all invasive cancers. Third, our study participants had a high recurrence-free survival rate (91 out of 94 patients). Therefore, we were unable to correlate radiogenomic data with actual clinical outcomes such as recurrence or survival rates. Finally, we performed statistical analysis based on the dichotomized MRI parameters instead of continuous ones in order to present clinically useful imaging criteria for predicting the prognosis and selecting optimal treatment in breast cancer patients. Statistical analysis using continuous variable has the advantage of reflecting all information given by the data [47], but it is difficult to provide clinically useful cut-off values or criteria. A limitation of this study is that the number of study participants is too small to present reliable clinical standards. Large study participants are needed to obtain reliable results in the near future.

## Conclusion

This radiogenomic study using MRI phenotypes assessed by BI-RADS lexicon and texture analysis and whole RNA-sequencing data revealed the differentially expressed genes according to MRI features in breast cancer subtypes based on ER and HER 2 status. This suggests that MRI features may help advance breast cancer treatment into more targeted and personalized form in the future. Further large-scale radiogenomic studies involving patients at various clinical stages and correlating with the actual clinical outcome should be performed to verify the results and identify whether they can be clinically useful.

## Supplementary Information

**Additional file 1: Table S1.** The MRI phenotypes of 95 breast cancers. A total of 62 qualitative and quantitative MRI phenotypes of 95 breast cancers are summarized. The quantitative texture parameters were dichotomized by the mean value for the 95 cancers..

**Additional file 2: Table S2.** Differentially expressed genes associated with breast cancer according to MRI phenotypes in histologic subgroups. There were 143 genes that were significantly upregulatedor downregulatedwith of *P* < 0.05 and *Q* < 0.1 according to the MRI phenotypes in subgroup analysis. Pseudogenes or unidentified genes were not included.

## Data Availability

The datasets used in the current study are available from the corresponding author on reasonable request.

## Abbreviations

TNBC: Triple-negative breast cancer
ER: Estrogen receptor
HER2: Human epidermal growth factor receptor 2
BI-RADS: Breast imaging-reporting and data system
T2: T2-weighted images
PrecontrastT1: Precontrast T1-weighted images
PostcontrastT1: Postcontrast T1-weighted images at the first phase of contrast injection
SSF: Spatial scale filter
SD: Standard deviation
Log2FC: Log twofold change
