# Supplementary material for: MRI-based breast cancer radiogenomics using RNA profiling: association with subtypes in a single-center prospective study
Source: Breast Cancer Res. 2023 Jun 30;25:79. doi: 10.1186/s13058-023-01668-7 (PMC10311893; doi:10.1186/s13058-023-01668-7)
Supplement: Supplementary file 1 — Additional file 1: Table S1. The MRI phenotypes of 95 breast cancers. A total of 62 qualitative and quantitative MRI phenotypes of 95 breast cancers are summarized. The quantitative texture parameters were dichotomized by the mean value for the 95 cancers.. [file 13058_2023_1668_MOESM1_ESM.docx]

**Additional file 1**

**Table S1.** The MRI phenotypes of 95 breast cancers

| MRI phenotype | Case group | Control group |
| --- | --- | --- |
| Tumor size | > 20 mm (60) | ≤ 20 mm (35) |
| Multiplicity | Multiple (28) | Single (67) |
| Morphology |  |  |
| Lesion type | Mass (87) | Non-mass enhancement (8) |
| Mass shape | Irregular (82) | Oval or round (5) |
| Mass margin | Spiculated (17) | Circumscribed or irregular (70) |
| Mass internal enhancement  characteristics | Rim (35) | Homogeneous or heterogeneous (52) |
| Non-mass enhancement distribution | Segmental (6) | Focal, linear, regional, diffuse (2) |
| Non-mass enhancement internal  enhancement pattern | Clustered ring, clumped (6) | Homogeneous, heterogeneous (2) |
| Texture analysis* |  |  |
| Mean on T2 (SSF 0) | > 220.05 (40) | ≤ 220.05 (55) |
| Mean on T2 (SSF2) | > 90.05 (43) | ≤ 90.05 (52) |
| Mean on T2 (SSF5) | > 222.60 (42) | ≤ 222.60 (53) |
| Standard deviation on T2 (SSF 0) | > 62.82 (28) | ≤ 62.82 (67) |
| Standard deviation on T2 (SSF 2) | > 180.84 (36) | ≤ 180.84 (59) |
| Standard deviation on T2 (SSF 5) | > 172.25 (29) | ≤ 172.25 (66) |
| Entropy on T2 (SSF 0) | > 5.00 (50) | ≤ 5.00 (45) |
| Entropy on T2 (SSF 2) | > 5.72 (51) | ≤ 5.72 (44) |
| Entropy on T2 (SSF 5) | > 5.67 (50) | ≤ 5.67 (45) |
| Mean of positive pixels on T2 (SSF 0) | > 220.05 (40) | ≤ 220.05 (55) |
| Mean of positive pixels on T2 (SSF 2) | > 182.38 (38) | ≤ 182.38 (57) |
| Mean of positive pixels on T2 (SSF 5) | > 263.90 (32) | ≤ 263.90 (63) |
| Skewness on T2 (SSF 0) | > l0.31l (72) | ≤ l0.31l (23) |
| Skewness on T2 (SSF 2) | > l0.33l (49) | ≤ l0.33l (46) |
| Skewness on T2 (SSF 5) | > l0.09l (80) | ≤ l0.09l (15) |
| Kurtosis on T2 (SSF 0) | ≤ 0.88 (64) | > 0.88 (31) |
| Kurtosis on T2 (SSF 2) | ≤ 0.72 (67) | > 0.72 (28) |
| Kurtosis on T2 (SSF 5) | ≤ -0.30 (65) | > -0.30 (30) |
| Mean on PrecontrastT1 (SSF 0) | ≤ 124.13 (51) | > 124.13 (44) |
| Mean on PrecontrastT1 (SSF 2) | ≤ 46.33 (58) | > 46.33 (37) |
| Mean on PrecontrastT1 (SSF 5) | ≤ 98.01 (55) | > 98.01 (40) |
| Standard deviation on PrecontrastT1 (SSF 0) | > 23.59 (38) | ≤ 23.59 (57) |
| Standard deviation on PrecontrastT1 (SSF 2) | > 78.66 (37) | ≤ 78.66 (58) |
| Standard deviation on PrecontrastT1 (SSF 5) | > 63.90 (32) | ≤ 63.90 (63) |
| Entropy on PrecontrastT1 (SSF 0) | > 4.10 (48) | ≤ 4.10 (47) |
| Entropy on PrecontrastT1 (SSF 2) | > 4.90 (51) | ≤ 4.90 (44) |
| Entropy on PrecontrastT1 (SSF 5) | > 4.81 (49) | ≤ 4.81 (46) |
| Mean of positive pixels on PrecontrastT1 (SSF 0) | ≤ 124.13 (51) | > 124.13 (44) |
| Mean of positive pixels on PrecontrastT1 (SSF 2) | ≤ 81.80 (57) | > 81.80 (38) |
| Mean of positive pixels on PrecontrastT1 (SSF 5) | ≤ 109.17 (55) | > 109.17 (40) |
| Skewness on PrecontrastT1 (SSF 0) | > l-0.69l (57) | ≤ l-0.69l (38) |
| Skewness on PrecontrastT1 (SSF 2) | > l0.23l (60) | ≤ l0.23l (35) |
| Skewness on PrecontrastT1 (SSF 5) | > l-0.13l (81) | ≤ l-0.13l (14) |
| Kurtosis on PrecontrastT1 (SSF 0) | ≤ 1.99 (59) | > 1.99 (36) |
| Kurtosis on PrecontrastT1 (SSF 2) | ≤ 0.78 (55) | > 0.78 (40) |
| Kurtosis on PrecontrastT1 (SSF 5) | ≤ -0.17 (53) | > -0.17 (42) |
| Mean on PrecontrastT1 (SSF 0) | > 264.87 (50) | ≤ 264.87 (45) |
| Mean on PostcontrastT1 (SSF 2) | > 161.71 (49) | ≤ 161.71 (46) |
| Mean on PrecontrastT1 (SSF 5) | > 369.45 (48) | ≤ 369.45 (47) |
| Standard deviation on PostcontrastT1 (SSF 0) | > 88.54 (41) | ≤ 88.54 (54) |
| Standard deviation on PostcontrastT1 (SSF 2) | > 279.56 (44) | ≤ 279.56 (51) |
| Standard deviation on PostcontrastT1 (SSF 5) | > 239.67 (38) | ≤ 239.67 (57) |
| Entropy on PostcontrastT1 (SSF 0) | > 4.93 (51) | ≤ 4.93 (44) |
| Entropy on PostcontrastT1 (SSF 2) | > 5.31 (51) | ≤ 5.31 (44) |
| Entropy on PostcontrastT1 (SSF 5) | > 5.27 (50) | ≤ 5.27 (45) |
| Mean of positive pixels on PostcontrastT1 (SSF 0) | > 264.87 (50) | ≤ 264.87 (45) |
| Mean of positive pixels on PostcontrastT1 (SSF 2) | > 303.63 (44) | ≤ 303.63 (51) |
| Mean of positive pixels on PostcontrastT1 (SSF 5) | > 408.07 (46) | ≤ 408.07 (49) |
| Skewness on PostcontrastT1 (SSF 0) | > l-0.29l (50) | ≤ l-0.29l (45) |
| Skewness on PostcontrastT1 (SSF 2) | > l-0.11l (63) | ≤ l-0.11l (32) |
| Skewness on PostcontrastT1 (SSF 5) | >l-0.07l (77) | ≤l-0.07l (18) |
| Kurtosis on PostcontrastT1 (SSF 0) | ≤ -0.24 (61) | > -0.24 (34) |
| Kurtosis on PostcontrastT1 (SSF 2) | ≤ -0.22 (50) | > -0.22 (45) |
| Kurtosis on PostcontrastT1 (SSF 5) | ≤ -0.56 (55) | > -0.56 (40) |

Data is the number of cancer in parentheses. T2 = T2-weighted images, PrecontrastT1 = precontrast T1-weighted images, PostcontrastT1 = postcontrast T1-weighted images at the first phase of contrast injection, SSF = spatial scale filter.

*The quantitative parameters were dichotomized by the mean value for the 95 cancers.
