# Supplementary material for: MRI-based breast cancer radiogenomics using RNA profiling: association with subtypes in a single-center prospective study
Source: Breast Cancer Res. 2023 Jun 30;25:79. doi: 10.1186/s13058-023-01668-7 (PMC10311893; doi:10.1186/s13058-023-01668-7)
Supplement: Supplementary file 2 — Additional file 2: Table S2. Differentially expressed genes associated with breast cancer according to MRI phenotypes in histologic subgroups. There were 143 genes that were significantly upregulatedor downregulatedwith of P < 0.05 and Q < 0.1 according to the MRI phenotypes in subgroup analysis. Pseudogenes or unidentified genes were not included. [file 13058_2023_1668_MOESM2_ESM.docx]

**Additional file 2**

**Table S2.** Differentially expressed genes associated with breast cancer according to MRI phenotypes in histologic subgroups

| Histologic subgroup | MRI phenotype | Genes | *Q* value | Log2FC | *P* value |
| --- | --- | --- | --- | --- | --- |
| ER-positive cancer | Lesion type | SNORA31 | .003 | 5.93 | < .001 |
|  |  | SNORD33 | .004 | 5.66 | < .001 |
|  |  | HIST2H3A | .016 | 5.38 | < .001 |
|  |  | *OR2A7* | .002 | 4.68 | < .001 |
|  |  | *CCL3L1* | .047 | 4.40 | .002 |
|  |  | SNORD10 | .013 | 4.37 | < .001 |
|  |  | *TBCA* | .019 | 4.12 | .001 |
|  |  | SNORA23 | .022 | 4.10 | .001 |
|  |  | *CCL4L2* | .023 | 4.09 | .001 |
|  |  | *NBPF16* | .011 | 4.02 | < .001 |
|  |  | *HLA-B* | .003 | 3.61 | < .001 |
|  |  | SNHG12 | .066 | 3.43 | .002 |
|  |  | *HLA-DRA* | .068 | 3.38 | .003 |
|  |  | *ARL17A* | .048 | 3.37 | .002 |
|  |  | *FTH1* | .001 | 3.03 | < .001 |
|  |  | *TUBA1B* | .035 | 3.00 | .001 |
|  |  | MIR206 | .053 | 2.86 | .002 |
|  |  | *NPIPA2* | .052 | 2.73 | .002 |
|  |  | *RPL10* | .027 | 2.69 | .001 |
|  |  | *SLC39A7* | .048 | 2.65 | .002 |
|  |  | *NPIPA7* | .089 | 2.65 | .004 |
|  |  | *BRD2* | .022 | 2.50 | .001 |
|  |  | *RPS16* | .053 | 2.45 | .002 |
|  |  | *GTF2I* | .077 | 2.38 | .003 |
|  |  | *CCL3* | .095 | 2.34 | .004 |
|  |  | *RPL29* | .064 | 2.32 | .002 |
|  |  | LINC01001 | .080 | 2.26 | .003 |
|  |  | *HMGN1* | .081 | 2.11 | .003 |
|  |  | *RPL18A* | .084 | 2.09 | .003 |
|  |  | *CD9* | .080 | 2.04 | .003 |
|  |  | *SSR2* | .034 | 2.01 | .001 |
|  |  | *DDX42* | .008 | -2.01 | < .001 |
|  |  | *KRTAP19-1* | < .001 | -2.09 | < .001 |
|  |  | *VCY* | .006 | -2.15 | < .001 |
|  |  | *CHD4* | .015 | -2.21 | < .001 |
|  |  | *OSTF1* | .063 | -2.22 | .002 |
|  |  | *SOX17* | .003 | -2.28 | < .001 |
|  |  | *PCDHB16* | .008 | -2.34 | < .001 |
|  |  | *GOLGA6C* | .046 | -2.36 | .002 |
|  |  | *KRTAP13-2* | < .001 | -2.39 | < .001 |
|  |  | SNORA30 | .044 | -2.44 | .001 |
|  |  | *SAPCD1* | < .001 | -2.47 | < .001 |
|  |  | *OR2B3* | .076 | -2.52 | .003 |
|  |  | *NTS* | < .001 | -2.71 | < .001 |
|  |  | *SPANXB1* | .030 | -2.91 | .001 |
|  |  | MIR648 | < .001 | -3.20 | < .001 |
|  |  | SNORD109A | .021 | -3.24 | .001 |
|  |  | MIR126 | < .001 | -3.63 | < .001 |
|  |  | MIR593 | < .001 | -3.91 | < .001 |
|  |  | *PPIAL4A* | .086 | -4.18 | .003 |
|  |  | *H2AFB2* | .021 | -5.78 | .001 |
|  |  | MIR597 | .001 | -8.05 | < .001 |
|  |  | MIR941-1 | .091 | -8.65 | .004 |
|  | Standard deviation on T2 (SSF 0) | *MUC2* | .012 | 2.16 | < .001 |
| ER-negative cancer | Lesion type | HIST2H4A | .063 | 3.21 | < .001 |
|  |  | *IGF1* | .049 | -2.09 | < .001 |
|  |  | *GNG11* | .063 | -3.17 | < .001 |
| HER2-positive cancer | Lesion type | *OR2M4* | .067 | -2.43 | < .001 |
|  |  | *OR10A6* | .021 | -2.46 | < .001 |
|  | Entropy on PostcontrastT1 (SSF 0) | *ZNF785* | .006 | -2.14 | < .001 |
|  |  | *VSTM2L* | .003 | -2.63 | < .001 |
|  |  | *OR6C3* | < .001 | -3.05 | < .001 |
|  |  | *TCHH* | .001 | -3.08 | < .001 |
|  | Entropy on PostcontrastT1 (SSF 2) | *OR10A3* | .007 | -2.65 | < .001 |
|  | Mean of positive pixels on PostcontrastT1 (SSF 2) | *POTEM* | .032 | 3.03 | < .001 |
|  |  | *MLKL* | .064 | 2.20 | < .001 |
|  | Entropy on PostcontrastT1 (SSF 5) | *NPW* | .060 | -3.68 | < .001 |
|  |  | *ARL17B* | .098 | -3.73 | < .001 |
|  | Mean of positive pixels on T2 (SSF 5) | *CXCL10* | .080 | -3.27 | < .001 |
| HER2-negative cancer | Lesion type | SNORA46 | .086 | 5.08 | .003 |
|  |  | SNORA3 | .088 | 4.79 | .003 |
|  |  | SNORA31 | .010 | 4.18 | < .001 |
|  |  | *CCL3L1* | .026 | 3.84 | .001 |
|  |  | SNORD10 | .094 | 2.77 | .003 |
|  |  | *TUBA1B* | .014 | 2.59 | < .001 |
|  |  | SCARNA12 | .037 | 2.41 | .001 |
|  |  | *BLOC1S4* | .007 | -2.09 | < .001 |
|  |  | *VCX3B* | .059 | -2.13 | .002 |
|  |  | MIR648 | .005 | -2.18 | < .001 |
|  |  | FAM27E4 | .058 | -2.28 | .002 |
|  |  | MIR616 | .005 | -2.40 | < .001 |
|  |  | *HP* | .029 | -2.45 | .001 |
|  |  | MIR126 | .006 | -2.46 | < .001 |
|  |  | *SUMO4* | .044 | -2.83 | .001 |
|  |  | MIR593 | .005 | -2.85 | < .001 |
|  |  | *FOXD4L3* | .051 | -3.70 | .001 |
| Triple-negative cancer | Skewness on PostcontrastT1 (SSF 0) | SNORA79 | 069 | -6.74 | .001 |
|  | Mean on PrecontrastT1 (SSF 0) | *SKI* | .086 | -2.10 | < .001 |
|  |  | *EN1* | .070 | -2.31 | < .001 |
|  |  | *KRT81* | .070 | -3.96 | < .001 |
|  |  | MIR1307 | .041 | -4.96 | < .001 |
|  | Mean of positive pixels on PrecontrastT1 (SSF 0) | *SKI* | .086 | -2.10 | < .001 |
|  |  | *EN1* | .070 | -2.31 | < .001 |
|  |  | *KRT81* | .070 | -3.96 | < .001 |
|  |  | MIR1307 | .041 | -4.96 | < .001 |
|  | Mean on PrecontrastT1 (SSF 2) | *OR2G3* | < .001 | -2.74 | < .001 |
|  | Mean of positive pixels on PostcontrastT1 (SSF 5) | VTRNA2-1 | .018 | -10.38 | < .001 |
|  | Mean on PrecontrastT1 (SSF 5) | *ZNF785* | .096 | -2.12 | < .001 |
|  |  | SERTAD4-AS1 | .072 | -2.16 | < .001 |
|  |  | *WBSCR22* | .057 | -2.23 | < .001 |
|  |  | *POLR1E* | .007 | -2.24 | < .001 |
|  |  | *FAM181B* | .046 | -2.26 | < .001 |
|  |  | *DHDDS* | .084 | -2.31 | < .001 |
|  |  | SLX1A-SULT1A3 | < .001 | -2.32 | < .001 |
|  |  | *DDX11* | .099 | -2.54 | < .001 |
|  |  | *GFOD1* | .046 | -2.60 | < .001 |
|  |  | *PCDHB11* | .046 | -2.89 | < .001 |
|  |  | *OR10A6* | .099 | -2.90 | < .001 |
|  |  | *ADRBK1* | .008 | -2.95 | < .001 |
|  |  | *P2RY8* | .099 | -3.26 | < .001 |
|  |  | *OR2G3* | .003 | -3.30 | < .001 |
|  |  | *AKAP17A* | .083 | -3.51 | < .001 |
|  |  | *BCL2* | .024 | -3.82 | < .001 |
|  | Standard deviation on PrecontrastT1 (SSF 5) | *H2AFB3* | .099 | 5.40 | .002 |
|  |  | *CLEC3A* | .036 | 4.50 | .001 |
|  |  | *SRGN* | .062 | 3.72 | .001 |
|  |  | *DACT1* | .002 | 3.61 | < .001 |
|  |  | *TAT* | .035 | 2.98 | < .001 |
|  |  | *CGA* | .035 | 2.89 | .001 |
|  |  | *PCDHB12* | .067 | 2.87 | .001 |
|  |  | *HSPG2* | .084 | 2.85 | .002 |
|  |  | *COX16* | .046 | 2.84 | .001 |
|  |  | *ALDH2* | .057 | 2.78 | .001 |
|  |  | *HOXC5* | .037 | 2.70 | .001 |
|  |  | *KERA* | < .001 | 2.44 | < .001 |
|  |  | *PRSS35* | .035 | 2.41 | < .001 |
|  |  | *ABCC5* | .007 | 2.36 | < .001 |
|  |  | *KMT2D* | .035 | 2.35 | < .001 |
|  |  | *OSGEPL1* | .073 | 2.34 | .002 |
|  |  | *INPP5E* | .035 | 2.30 | < .001 |
|  |  | *FBP1* | .035 | 2.29 | < .001 |
|  |  | *VMP1* | .037 | 2.26 | .001 |
|  |  | *OR10X1* | .008 | 2.24 | < .001 |
|  |  | *SULT2B1* | .035 | 2.19 | < .001 |
|  |  | *POTED* | .063 | 2.16 | .001 |
|  |  | *CCDC30* | .076 | 2.13 | .002 |
|  |  | *RPL7A* | .089 | 2.09 | .002 |
|  |  | *ALKBH2* | .096 | 2.09 | .002 |
|  |  | LL0XNC01-250H12.3 | .035 | 2.07 | < .001 |
|  |  | *FZD2* | .085 | 2.06 | .002 |
|  |  | *MTG1* | .049 | 2.04 | .001 |
|  |  | *HMG20B* | .055 | 2.04 | .001 |
|  |  | *ZNF99* | .035 | 2.01 | < .001 |
|  |  | *HNRNPA1P48* | .041 | -2.55 | .001 |
|  |  | *PRDX4* | .094 | -2.80 | .002 |
|  |  | *RPL28* | .035 | -3.05 | < .001 |
|  |  | *IRF9* | .100 | -3.08 | .002 |
|  |  | *NOP10* | .052 | -3.45 | .001 |
|  |  | *RPS11* | .049 | -3.76 | .001 |
|  |  | *CCL4L2* | .035 | -4.01 | < .001 |
|  |  | SNORA46 | .035 | -5.90 | .001 |
|  |  | *IGLV4-69* | .091 | -6.03 | .002 |
|  |  | *IGLC2* | .016 | -6.18 | < .001 |
|  |  | *IGKV1-33* | .067 | -6.34 | .001 |
|  |  | *IGLV2-14* | .035 | -6.66 | .001 |
|  |  | *IGLV2-11* | .035 | -6.95 | < .001 |
|  |  | SNORA50 | .063 | -9.25 | .001 |

There were 143 genes that were significantly upregulated (log2FC > 2.0) or downregulated (log2FC < –2.0) with of *P* < .05 and *Q* < .1 according to the MRI phenotypes in subgroup analysis. Pseudogenes or unidentified genes were not included. Log2FC = log 2-fold change, PrecontrastT1 = precontrast T1-weighted images, PostcontrastT1 = postcontrast T1-weighted images at the first phase of contrast injection, T2 = T2-weighted images, SSF = spatial scale filter, ER = estrogen receptor, HER2 = human epidermal growth factor receptor 2.
